# Biological Characteristics of *Dasineura jujubifolia* and Its Parasitoid Natural Enemies in Hami Region of Xinjiang (China)

**DOI:** 10.3390/insects16111118

**Published:** 2025-10-31

**Authors:** Kailiang Li, Zhiqiang Ge, Zhenyu Zhang, Yuhao Nie, Hongying Hu

**Affiliations:** Xinjiang Key Laboratory of Biological Resources and Genetic Engineering, College of Life Science and Technology, Xinjiang University, Urumqi 830017, China; 107556522146@stu.xju.edu.cn (K.L.); 107552301046@stu.xju.edu.cn (Z.G.); james_cotton@163.com (Z.Z.); nieyuhaoxj@163.com (Y.N.)

**Keywords:** *Dasineura jujubifolia*, parasitic natural enemies, occurrence patterns, *Pseudotorymus samsatensis*, natural enemy insect conservation

## Abstract

**Simple Summary:**

The jujube gall midge *Dasineura jujubifolia* induces leaf galls that weaken trees and reduce yield in Xinjiang’s arid jujube orchards, highlighting an urgent need for region-specific, evidence-based management. We carried out a season-long, standardized field survey in two orchards in Hami (Xinjiang, China), combined with gall dissection and laboratory rearing, to document the midge’s biology and associated natural enemies. The midge completed 4–5 generations annually, and we recorded five parasitoid wasps, including two species newly recorded in China—*Pseudotorymus samsatensis* and *Baryscapus adalia*. These natural enemies exhibited distinct parasitism strategies; *P. samsatensis* was dominant and closely tracked host population peaks. Laboratory assays clarified daily emergence rhythms, adult longevity (under different diets), and sex ratios, providing practical parameters to optimize parasitoid conservation and reduced-pesticide management in jujube orchards.

**Abstract:**

Severe leaf galling by the jujube gall midge *Dasineura jujubifolia* (Diptera: Cecidomyiidae) compromises photosynthesis and yield in arid-zone jujube orchards, yet Xinjiang-specific evidence to guide biological control has been scarce. Here we provide the first systematic characterization in Xinjiang (Hami, China) of *D. jujubifolia* and its parasitoid complex, integrating region-specific field surveys with gall dissection and laboratory assays. We documented five parasitoid wasps, including two species newly recorded in China—*Pseudotorymus samsatensis* (Hymenoptera: Torymidae) and *Baryscapus adalia* (Hymenoptera: Eulophidae). In Hami, the host completed 4–5 generations per year with a 19–24-day generation time. Functional roles were partitioned: *P. samsatensis* (dominant), *Systasis parvula* (Hymenoptera: Pteromalidae), and *B. adalia* were larval ectoparasitoids, whereas *Aprostocetus* sp. (Hymenoptera: Eulophidae) and *Synopeas* sp. (Hymenoptera: Platygastridae) were endoparasitoids. Time-series data revealed tight temporal synchrony between *P. samsatensis* and host peaks. Controlled experiments quantified daily emergence rhythms, diet-dependent adult longevity, and sex ratios, providing parameters to inform release timing and conservation in biological control programs. Collectively, these findings establish management-ready baselines for *D. jujubifolia* and its parasitoids in arid jujube systems and support conservation-oriented, reduced-pesticide integrated pest management (IPM).

## 1. Introduction

*Ziziphus jujuba* Mill. (Rosales: Rhamnaceae) has been cultivated for more than 4000 years in the middle and lower reaches of the Yellow River basin in China. Cultivation has since expanded to other regions of China, to neighbouring areas of Asia, and further to Australia, North Africa, Southern Europe, the Middle East, and the southwestern United States [1,2]. As a key economic crop in arid and semi-arid regions worldwide, the jujube tree plays an irreplaceable role in mitigating soil desertification and delivering essential ecosystem services. It possesses significant ecological value as well as considerable nutritional and medicinal importance [3,4,5]. Currently, more than 700 jujube varieties are cultivated in China, which ranks first worldwide in both cultivation area and yield. Furthermore, *Z. jujuba* plays a multidimensional role in international trade, cultural exchange, and the promotion of global health [2,6].

*Dasineura jujubifolia* (Jiao & Bu, 2017) (Diptera, Cecidomyiidae, *Dasineura*) is recognized as an economically important pest of *Z. jujuba* [7]. This species was first recorded in Shandong Province, China, in 1966, and has since caused persistent damage across major jujube-growing regions in China. The larvae feed on tender leaves, inducing the formation of galls that result in leaf distortion, desiccation, and eventual abscission. Such damage severely impairs photosynthesis and plant growth [8]. Infestation is estimated to reduce yields by at least 20%. In China, *D. jujubifolia* has emerged as one of the principal pests in jujube orchards, with infestation rates reaching up to 100% in some regions, posing a serious threat to local production [9]. The species was formally described in 2017 based on its morphological characteristics [7]. To date, the occurrence of *D. jujubifolia* has been confirmed only in China and South Korea. In South Korea, it was first detected in 2011, where it rapidly established and spread throughout major jujube production areas, becoming a serious invasive pest threatening local jujube cultivation [10]. In addition to *D. jujubifolia*, three other gall midge species have been reported globally as important leaf pests of jujube, namely, *Phyllodiplosis jujubae* (Grover & Bakhshi, 1978) (Diptera: Cecidomyiidae) [11], *Silvestrina jujubae* (Chandra, 1988) (Diptera: Cecidomyiidae) [12], and *Asphondylia singanallurensis* (Vasanthakumar & Sharma, 2020) (Diptera: Cecidomyiidae) [13]. Among them, *P. jujubae* exhibits similarities with *D. jujubifolia* in damage symptoms, particularly in inducing leaf-rolling galls on jujube foliage. However, the two species are distinguished by gall coloration: galls induced by *P. jujubae* remain pale or weakly pigmented, whereas those formed by *D. jujubifolia* are typically dark purplish-red—an important diagnostic feature for species differentiation.

Chemical control still predominates against *D. jujubifolia*, yet prolonged or excessive applications select for resistance and erode efficacy [14,15]. Broad-spectrum activity and residue accumulation disrupt natural-enemy communities, soil and aquatic ecosystems, and pollinators, triggering secondary pest outbreaks and weakening agroecosystem self-regulation [16,17,18,19]. By contrast, biological control provides environmentally safe, durable suppression and a sustainable pathway to integrated management [20,21,22,23]. Among bioagents, parasitoid wasps are central because they directly suppress hosts and can synchronize with host phenology. Their performance, however, is highly context-dependent, varying with species diversity, behavioral ecology (foraging/oviposition), host specificity, and management/landscape settings [24,25,26,27,28]. Accordingly, a unified “species–mechanism–application” program is essential: identify dominant parasitoids and attack modes; quantify development, fecundity, longevity, phenology, and climatic responses; and evaluate pesticide selectivity and habitat manipulation to enable conservation biological control. On this basis, a parasitoid-centred ecological strategy should integrate agronomic practices (pruning, tillage, mulching), physical barriers, and conservation/augmentative releases, with monitoring–early warning to time interventions—reducing chemical inputs while achieving robust suppression and ecological balance.

Research on parasitoids of *D. jujubifolia* remains in its infancy, with only sporadic reports of parasitism and limited efforts at species-level identification. For instance, an ectoparasitoid in the family Pteromalidae was reported in Shandong Province, China [8]. In another report, Zhang (1994) documented two parasitoid species—*Systasis* sp. and *Pseudotorymus* sp.—from jujube orchards in Changli County, Hebei Province, and provided brief morphological descriptions [29]. Liu (2023) observed three parasitoid species in the Alar region of Xinjiang, including one ectoparasitoid and two endoparasitoids, although their precise taxonomic identities remain unresolved [30]. Although these studies provide valuable baseline information, the parasitoid community associated with *D. jujubifolia* remains poorly characterized, and systematic taxonomic research is lacking. Key biological parameters—including parasitism rate, parasitic strategy, and sex ratio—have yet to be determined. Comprehensive investigations into these traits are critical for developing evidence-based biological control strategies and for elucidating the ecological roles of parasitoids in natural ecosystems.

A systematic investigation of *D. jujubifolia* and its associated parasitoids was conducted in the Hami region of Xinjiang, China, between September 2020 and October 2021. Through field sampling, laboratory rearing, and taxonomic identification, we examined key biological traits, including species composition, morphology, population dynamics, parasitism strategies, and the longevity and sex ratio of adult parasitoids. These results establish a scientific basis for understanding the population dynamics of *D. jujubifolia* and the regulatory role of its natural enemies, and provide essential baseline data for the development of future integrated pest management strategies.

## 2. Materials and Methods

### 2.1. Study Sites

This study was conducted in two representative jujube orchards in Huicheng Township, Yizhou District, Hami City, Xinjiang, China. The geographic coordinates and elevations of the two orchards were as follows: Orchard A (42°48′21.7512″ N, 93°21′34.2144″ E; elevation: 681 m) and Orchard B (42°50′23.5248″ N, 93°21′30.4524″ E; elevation: 687 m). Both orchards were planted with the Hami Junzao cultivar. The trees were approximately 10–11 years old and managed with uniform irrigation, fertilization, and pest control practices. The primary difference lay in their spatial configuration; Orchard A covered a smaller area (2.67 ha) with a denser planting pattern (intra-row spacing, 89 ± 14 cm; inter-row spacing, 296 ± 11 cm), whereas Orchard B covered a larger area (3 ha) with a more spacious planting arrangement (102 ± 13 cm × 345 ± 27 cm). Despite these spatial differences, the phenological stages of the jujube trees were consistent across both orchards. The trees exhibited vigorous growth, with budbreak in mid-April and flowering beginning in late May. Full bloom occurred in early June, concurrent with fruit set. Flowering gradually declined by late June, though some blossoms persisted until late July or early August. Fruit ripened from mid- to late September, and leaf abscission began in early October, marking the onset of the harvest period.

### 2.2. Life History and Damage Symptoms of D. jujubifolia

We combined field observations and laboratory rearing to systematically investigate the life history and damage symptoms of *D. jujubifolia*. Newly emerged jujube leaves were examined for *D. jujubifolia* eggs. When detected, infested branches were enclosed in 100-mesh nylon bags and monitored daily. The developmental duration and damage symptoms of each life stage were recorded. In addition, galls at different developmental stages were collected and brought to the laboratory for dissection and rearing. Larval damage at different instars was documented, and images were captured with a Nikon SMZ25 stereomicroscope (Nikon Corporation, Tokyo, Japan). In the laboratory, newly emerged adults were introduced to water-cultured jujube shoots to observe oviposition and larval hatching behavior. Rearing conditions were maintained at 35 ± 5% RH and 28 ± 3 °C. Between April 20 and September 10, 2021, systematic sweep net sampling (mesh size: 0.5 mm) was conducted every 10 days in both orchards to monitor adult populations. Sweeping was performed around jujube trees, with the number of sweeps at each site set to one-tenth of the number of tree rows. All collected specimens were sealed in plastic bags and preserved in 75% ethanol. Environmental variables, including collection date, elevation, temperature, and humidity, were recorded. Additionally, a Malaise trap was installed at Site A, with trap bottles replaced every 10 days. Data from the Malaise trap and sweep net samples were analyzed together to determine the annual life cycle of the species.

### 2.3. Parasitoid Species, Parasitism Modes, and Rates of D. jujubifolia

We employed a systematic five-point sampling strategy to investigate parasitoid species associated with *D. jujubifolia*, as well as their parasitism modes and rates. Ten jujube trees were randomly selected and marked across two orchards. Because gall occurrence at the tree level was random (any newly flushed shoot could be attacked), a five-point sampling scheme (center and four cardinal directions) was used in each orchard, with one randomly selected tree at each point (five trees per orchard and ten in total across two orchards). Each tree was divided into eight sampling zones, corresponding to the upper and lower canopy layers in the four cardinal directions (east, south, west, and north). From each sampling zone, one mid-developing gall containing second- to third-instar larvae of *D. jujubifolia* was randomly collected, resulting in a total of 80 galls per sampling event. Sampling was conducted at 10-day intervals between May 10 and August 30. To avoid interference with natural parasitism, no chemical pesticides were applied in either orchard during the sampling period. All galls were transported to the laboratory for dissection, where parasitism status was assessed under a dissecting microscope, and parasitoid species and modes of parasitism were recorded. Ectoparasitoid larvae obtained from dissections were reared under laboratory conditions, and their morphological traits and developmental stages were documented. Endoparasitoid observations were conducted by transferring unparasitized larvae from dissected galls into Petri dishes, enabling the direct monitoring and documentation of the parasitism process. Parasitism rates were calculated and expressed as the percentage of total larvae examined in the field samples. For detailed estimation, rates were calculated separately for ectoparasitoids (based on the number of larvae killed) and endoparasitoids (based on the number of adults that emerged). To identify the dominant parasitoid, we used the total number of host larvae killed as the sole criterion. For each species, we summed the number of larvae it killed across all sample units (galls, plants, or plots) to obtain its total hosts killed; the species with the largest total hosts killed was designated the dominant parasitoid. For ectoparasitoids, counts were based on larvae killed per gall verified by dissection or rearing; for endoparasitoids, each successfully emerged adult was counted as one host killed (1:1). To prevent duplicate counts, any galls in which parasitism could not be confidently assigned to a single species were excluded from the analysis.

### 2.4. Emergence, Longevity, and Sex Ratio of Parasitoid Adults

At adult emergence, both emergence behavior and daily emergence rhythm were recorded, with 50 individuals of each sex per species observed (*n* = 50 per sex per species). The laboratory environment was maintained at 28 ± 3 °C and 35 ± 5% RH. In the longevity assay, 30 adults of each sex per species were individually reared under two feeding treatments (15% honey solution and distilled water) in 15 × 90 mm glass tubes sealed with a small cotton plug (*n* = 30 per sex per feeding condition). Water or honey solution was applied daily to the cotton plug with a syringe, and the cotton was replaced every three days to maintain concentration stability and prevent mould growth. Adult longevity under different feeding conditions was recorded and summarized as mean ± standard error (mean ± SE).

### 2.5. Data Analysis

Statistical analyses were conducted in IBM SPSS Statistics version 30.0 at a significance level of α = 0.05. A two-way ANOVA was used to assess the effects of parasitoid species and feeding condition on adult longevity for each sex, followed by Tukey’s HSD test for post hoc comparisons. Results are presented as mean ± SE. Differences among species and between feeding conditions are indicated by different lowercase and uppercase letters, respectively. Morphological characteristics of insects were documented using a Nikon SMZ25 stereomicroscope and a Nikon Eclipse microscope equipped with a DS-U3 imaging system (Nikon Corporation, Tokyo, Japan). Body length of both females and males was measured from the vertex to the apex of the abdomen, excluding the antennae and ovipositor. Additional statistical analyses and figure generation were performed using Microsoft Excel and Origin 2024. Selected images were edited using Adobe Photoshop 2022.

## 3. Results

Across all metrics examined, we found no significant differences between Orchard A and Orchard B (all *p* > 0.05). Developmental durations of *D*. *jujubifolia* (egg, larval, pupal, and adult stages), host abundance metrics (larvae per gall and total larvae sampled), overall and guild-specific parasitism rates (ectoparasitoids vs. endoparasitoids), and parasitoid adult longevity and sex ratio were statistically comparable between orchards. Accordingly, to increase statistical power and better represent regional patterns, data from the two orchards were pooled for all subsequent analyses; orchard-wise summary statistics (mean ± SD) are provided in Table S1.

### 3.1. Life History of D. jujubifolia

In Hami, Xinjiang, *D. jujubifolia* completed one generation within 19–24 days, with the egg stage lasting 3.07 ± 0.30 days (mean ± SD), the larval stage 11.60 ± 1.39 days, the pupal stage 5.60 ± 1.10 days, and the adult stage 1.71 ± 0.49 days. In local jujube orchards, the species completes 4–5 generations per year.

**Figure 1:**
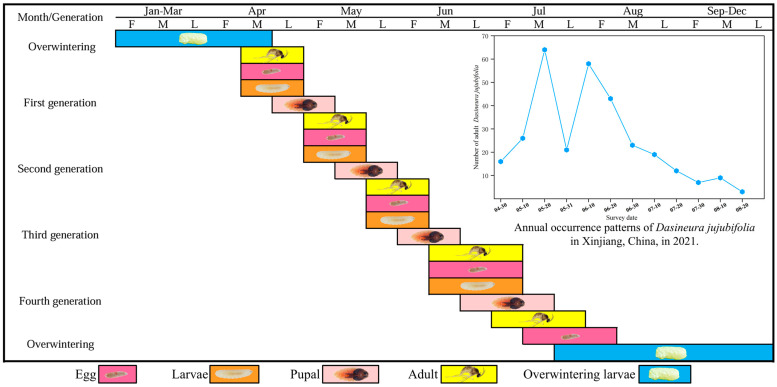
Population dynamics and annual life cycle of *D*. *jujubifolia* in Hami, Xinjiang, China (2021).

The overwintering generation of D. jujubifolia emerges as adults in mid-to-late April, representing the first population peak. Subsequent peaks occur in mid-May, early June, and mid-June, after which population density declines sharply. Although adults persist until August, their abundance continues to decrease. Owing to overlapping generations, the final generation of larvae burrows 2–5 cm into the soil by mid-to-late August to pupate and overwinter. Among the multiple generations, the first and second exhibit the highest population densities (Figure 1).

To verify whether *D. jujubifolia* could complete its life cycle under laboratory conditions, third-instar larvae were collected from the field and reared under controlled conditions (26 ± 2 °C, 60 ± 5% RH, 16L:8D). Larvae were provided with fresh jujube leaves or transferred to potted jujube plants grown indoors. They successfully pupated and produced adults that could mate and oviposit. However, first-instar larvae hatched in the laboratory failed to induce gall formation and gradually died, even when provided with tender leaves. Repeated trials under varied humidity and light conditions all failed, indicating that gall initiation and larval development are tightly coupled and depend on the natural gall microenvironment.

### 3.2. Morphological Characteristics of D. jujubifolia at Different Developmental Stages

**Figure 2:**
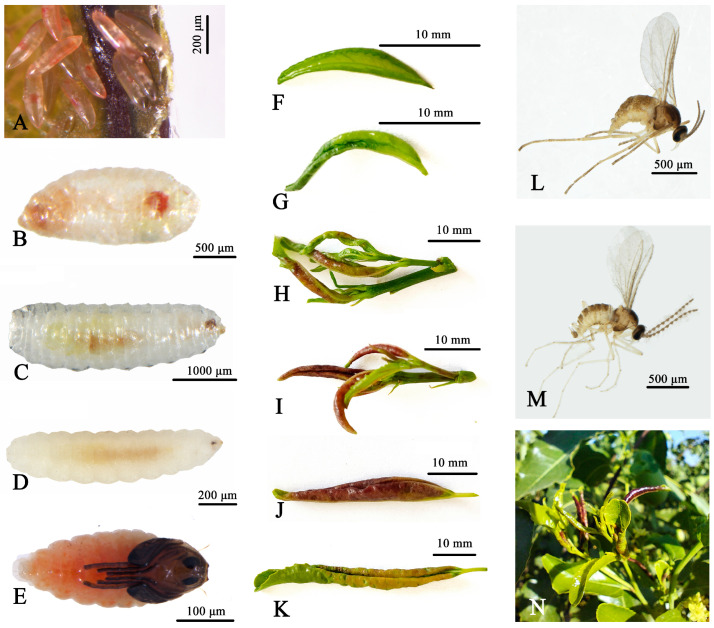
Morphological characteristics at different developmental stages and associated damage symptoms of *D*. *jujubifolia*. (**A**): Egg. (**B**): First-instar larva. (**C**): Second-instar larva. (**D**): Third-instar larva. (**E**): Pupa. (**F**): Healthy tender leaf. (**G**–**K**,**N**): Damage symptoms. (**L**): Female. (**M**): Male.

Egg: Elongate-oval, measuring 0.27–0.30 mm in length and 0.08–0.10 mm in width. At deposition, they are entirely transparent with a faint reddish hue. One end tapers, and the surface is enveloped in a transparent, glossy gelatinous layer. During development, a ring of punctate white lipid deposits forms beneath the surface, while the gloss gradually fades. The tapered end develops into the larval posterior, whereas the opposite end forms the head (Figure 2A).

Larvae: Early-instar larvae (L1) are fully transparent, 0.30–0.35 mm long, with visible antennae and active crawling movements (Figure 2B,C). They feed primarily on leaf sap adjacent to veins. Mature larvae (L3) are milky white, with distinctly segmented bodies and a characteristic Y-shaped sternal spatula on the ventral abdomen. Each body segment bears paired spiniform projections along the lateral margins, and body length ranges from 1.70 to 2.80 mm (Figure 2D).

Pupa: Exarate, 1.75–2.30 mm long, initially milky white, then turning yellowish-brown and finally opaque orange-red. A conspicuous pair of spines arises from the vertex. Cephalic setae are long, slender, and filamentous. Antennae, legs, and wing buds are well defined, with rounded antennal bases. The prothoracic spiracle is stout and blunt, tapering slightly distally. The abdomen consists of eight segments (Figure 2E).

Adult: Antennae are dark grey, and compound eyes black. The head and thorax are brown, and the abdomen light reddish-brown. Females measure 1.7–2.2 mm in body length, with antennae 0.9–1.1 mm long, extending beyond half the body length (Figure 2L). Female antennae comprise 15 segments, with a conical scape and ellipsoidal pedicel; the apical three flagellomeres are longer than the proximal ones. Wings are transparent, densely setose, and bear a few scales. The abdomen has eight segments, with sparse long setae distributed on the distal third of the terminal segment. Males are 1.6–1.8 mm long, with antennae 0.7–0.8 mm long, consisting of 14–15 segments. The clasping organ has a robust basal segment with sparse long setae dorsally and ventrally. Other characteristics resemble those of females (Figure 2M).

### 3.3. Damage Characteristics of D. jujubifolia

Larvae of *D. jujubifolia* inflict significant injury on *Ziziphus jujuba* by feeding on tender leaves and inducing gall formation. In the early stages, galls are bright green, later turning purplish-red and ultimately fading to yellow-green or desiccated prior to abscission. Between May and August 2021, we conducted systematic field surveys in more than 70 jujube orchards throughout Hami and adjacent areas (Xinjiang, China). The pest was detected in every surveyed orchard, and nearly all trees were infested, with an overall infestation rate of approximately 100%. The number of galls per tree typically ranged from 50 to 120. Severe galling suppresses new shoot growth and reduces the photosynthetic capacity of affected leaves, thereby impairing tree development and fruit yield (Figure 2N).

Early stage: Larvae feed on tender leaves, preventing normal expansion and inducing the inward curling of leaf margins to form cylindrical galls. They consume sap from internal tissues. When galls reach ~2 cm in length, they usually contain first- or second-instar larvae hatched from *D. jujubifolia* eggs (Figure 2G). As galls develop a faint purplish-red hue (Figure 2H), most larvae moult to the second instar.

Mid-stage: As the purplish-red coloration deepens (Figure 2I), third-instar larvae predominate. Each gall generally contains 2–20 larvae, with a maximum of 94 individuals recorded in a single gall.

Late stage: With the further intensification of the purplish-red colour (Figure 2J), petioles become fragile and detach under slight force. After abscission, larvae typically exit and burrow into the soil to pupate and later emerge as adults. In rare cases, pupation and adult emergence occur within the gall. If galls remain attached, larvae still exit and drop to the soil. Once the gall turns yellow-green (Figure 2K), it is vacant, often containing parasitoid pupae or exuviae of parasitized larvae. Galled leaves that do not abscise fail to grow properly and remain curled.

### 3.4. Morphological Characteristics and Parasitism Modes of Parasitoid Natural Enemies of D. jujubifolia

**Figure 3:**
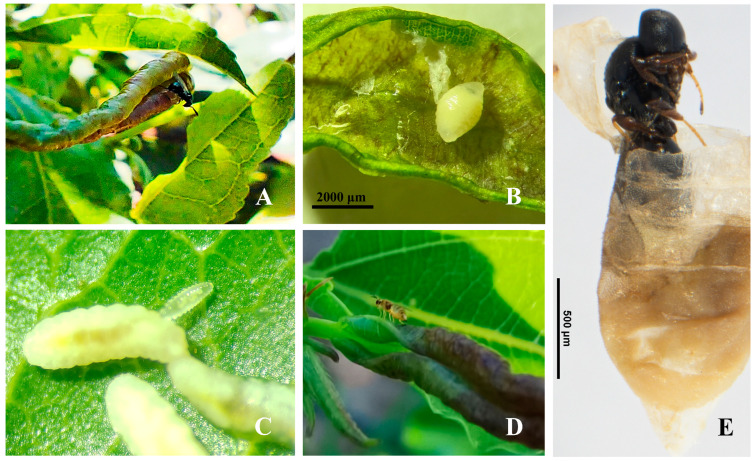
Modes of parasitism by natural enemies of *D. jujubifolia*. (**A**): *P. samsatensis* (ectoparasitoid) ovipositing through the gall wall. (**B**): *S. parvula* larva feeding externally on the host larva within the gall chamber. (**C**): A single *B. adalia* larva developing ectoparasitically on the body surface of a *D. jujubifolia* larva. (**D**): *Aprostocetus* sp. adult ovipositing on the gall surface (endoparasitoid). (**E**): *Synopeas* sp. adult recovered from a dissected gall.

Field investigations identified five parasitoid species associated with *D. jujubifolia*, with *Pseudotorymus* Doganlar (Hymenoptera: Torymidae) and *Baryscapus adalia* (Walker) (Hymenoptera: Eulophidae) newly recorded in China. *P. samsatensis* (Figure 3A), *Systasis parvula* (Thomson) (Hymenoptera: Pteromalidae) (Figure 3B), and *B. adalia* (Figure 3C) are ectoparasitoids of the larval stage, causing differential host mortality within galls. Lethality varied among species; *P. samsatensis* caused the highest mortality (4–19 larvae per gall, mean 9.15 ± 4.01), followed by *S. parvula* (2–17 larvae, mean 8.35 ± 3.49), whereas *B. adalia* exhibited lower lethality (2–9 larvae, mean 5.68 ± 1.93). *Aprostocetus* sp. (Hymenoptera: Eulophidae) (Figure 3D) and *Synopeas* sp. (Hymenoptera: Platygasteridae) (Figure 3E) were identified as endoparasitoids of the larval stage. Compared with non-parasitized cohorts, endoparasitoid emergence was delayed by ~2–5 days. Brief species descriptions are provided in the following section.

#### 3.4.1. *Pseudotorymus samsatensis* Doganlar

**Figure 4:**
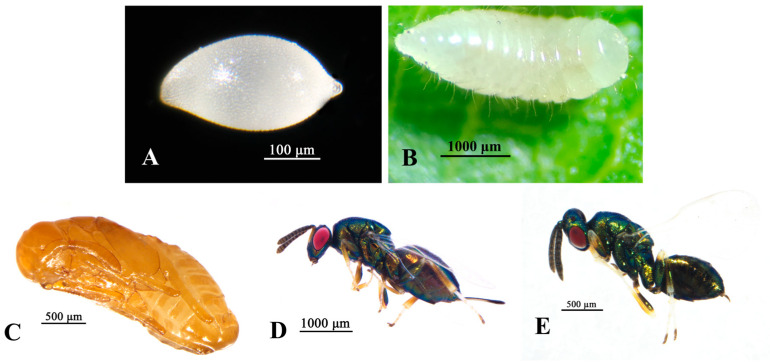
Dominant parasitoid of *D*. *jujubifolia—P*. *samsatensis*. (**A**): Egg. (**B**): Larva. (**C**): Pupa. (**D**): Female. (**E**): Male.

Eggs: Milky white in coloration, fusiform in shape (with pointed anterior and posterior tips with enlarged postero-medial portion), with densely punctate surfaces. Mean dimensions measure 0.31 mm (major axis) × 0.16 mm (minor axis) (Figure 4A).

Larvae: Early-instar larvae (L1) translucent throughout, body length 0.32–0.37 mm. Late-instar larvae (L3) are fusiform in shape, milky white and semi-translucent, with a smooth and lustrous cuticle; the body exhibits distinct segmentation with shallow intersegmental constrictions, comprising 13 somites, where the head segment is small and the terminal segment is nearly tubular; each segment is sparsely setose (Figure 4B).

Pupae: The newly formed pupa is whitish with no pigmentation, and subsequently the body coloration transitions through yellowish before finally darkening to black. The eyes undergo a chromatic shift from white to red. Prior to eclosion, the pupal integument develops a distinct greenish lustrous, while the pupal case becomes pale yellowish and semi-translucent. Sexual dimorphism is evident, with female pupae being significantly larger than their male counterparts, the ovipositor curved dorsally and folded along the abdominal. During the pupal stage, the oral organs, forelimbs, hindlimbs, and wings are clearly visible. The pupal stage exhibits clearly discernible morphological structures including mouthparts, forelegs, hindlegs, and wing (Figure 4C).

Adult: Female, body length 1.95–3.10 mm, metallic green with lustre. Head finely reticulate, antenna inserted center of face, antennal formula 11173. Mesoscutum with regular and prominent reticulation, notaulus deep and complete, propodeum without median carina, basal area with distinct but incomplete carinae, central area smooth. Mt1 to Mt4 each bearing a median posterior emargination; ovipositor clearly extending beyond the metasoma, 0.66× length of gaster (Figure 4D). Male, body length 1.53–2.23 mm. Otherwise similar to female (Figure 4E).

Distribution: China (Xinjiang) and Türkiye [31].

Hosts: *Dasineura jujubifolia*.

#### 3.4.2. *Systasis parvula* Thomson

**Figure 5:**
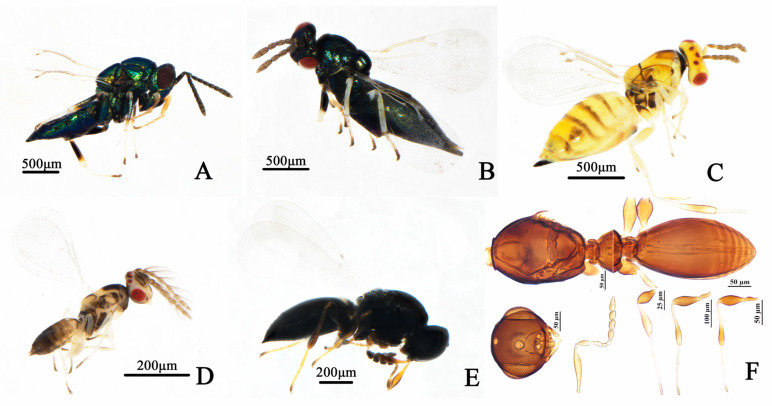
Parasitic natural enemies of *D*. *jujubifolia*. (**A**): *S. parvula* female. (**B**): *B*. *adalia* female. (**C**): *Aprostocetus* sp. female. (**D**): *Aprostocetus* sp. male. (**E**): *Synopeas* sp. female. (**F**): *Synopeas* sp. male.

Female, body length 1.68–2.45 mm, metallic green; antenna dark brown, scape metallic green with lustre (Figure 5A). In dorsal view, head and metapleuron with rough reticulate. Scape extending to level of anterior ocellus, 2/3 length of eye height; flagellum gradually widening; funicle with all segments approximate square; combined length of pedicel and flagellum subequal in width to head. Forewing with speculum, marginal vein significantly longer than postmarginal vein. Male body length 1.32–1.82 mm. Otherwise similar to female.

Distribution: China (Beijing, Hebei, Jilin, Heilongjiang, Shandong, Henan, Hunan, Hainan, Shaanxi, Xinjiang), Ireland, Sweden, Czech Republic, Slovakia, United Kingdom, Kazakhstan, and Hungary [32].

Hosts: *Bremiola caraganae*, *Dasineura herteroae*, *Schizomyia galiorum* and *Dasineura jujubifolia*.

#### 3.4.3. *Baryscapus adalia* (Walker)

Female, body length 1.83–2.19 mm, blackish green with lustre, eyes and ocellus red, antenna, femur dark brown, tibia yellowish. Antenna attached to the line of the lower ocular, antennal formula 11133; malar sulcus slightly curved; pronotum narrow; propodeum with median carina. Forewing with three setae on the dorsal surface of the submarginal vein, marginal vein 2.0× length of stigmal vein, postmarginal vein degenerated (Figure 5B). Male, unknown.

Distribution: China (Xinjiang), Bulgaria, Czech Republic, Slovakia, France, Germany, Hungary, Italy, Russia, Serbia, Sweden, Türkiye, and United Kingdom [33].

Hosts: *Dasineura jujubifolia*.

#### 3.4.4. *Aprostocetus* sp.

Female, body length 1.01–1.46 mm, blackish green with lustre, goose yellow with small brown spots. Malar sulcus V-shaped and slightly curved; anterior margin of clypeus with 2 distinct grooves; mandible 4-dentate; antenna with three-segmented flagellum and three-segmented clava. Pronotum narrow; notaulus complete; scutellum with conspicuous submedian groove and sublateral grooves; propodeum with median carina (Figure 5C). Male, body length 1.01–1.11 mm. Mandible three-dentate; antenna with four-segmented flagellum; propodeum and metasoma dark brown. Otherwise, similar to female (Figure 5D).

Distribution: worldwide.

Hosts: *Dasineura jujubifolia*

#### 3.4.5. *Synopeas* sp.

Female, body length 0.9–1.0 mm, black; tibia deep reddish-brown and tarsus light brown. Head vertex with slightly reticulate and leathery; upper face glabrous; antenna attached to the clypeus. Mesonotum smooth and covered with sparsely distributed setae, mesopleuron smooth. Metasoma 0.9–1.0× combined length of head and mesosoma, equal width to the mesosoma (Figure 5E). Male, body length 0.9–1.0 mm. Otherwise similar to female (Figure 5F).

Distribution: worldwide

Hosts: *Dasineura jujubifolia*.

### 3.5. Parasitism Rates and Community Dynamics of Parasitoids

Field surveys have shown that *P*. *samsatensis* was the dominant parasitoid of *D*. *jujubifolia*, at a natural parasitism rate of 13.6%. Parasitism rates of other species varied, with *S*. *parvula* and *B*. *adalia* exhibiting rates of 2.7% and 7.1%, respectively. Among endoparasitoids, *Aprostocetus* sp. had a relatively high rate of 8.9%, while *Synopeas* sp. accounted for 6.6%. When all parasitoid species were considered together, the overall parasitism rate of parasitoid natural enemies of *D. jujubifolia* was approximately 39.0%, with ectoparasitoids and endoparasitoids contributing 23.4% and 15.5%, respectively. This indicates that the parasitoid community exerts a strong collective regulatory effect on host populations in the field.

**Figure 6:**
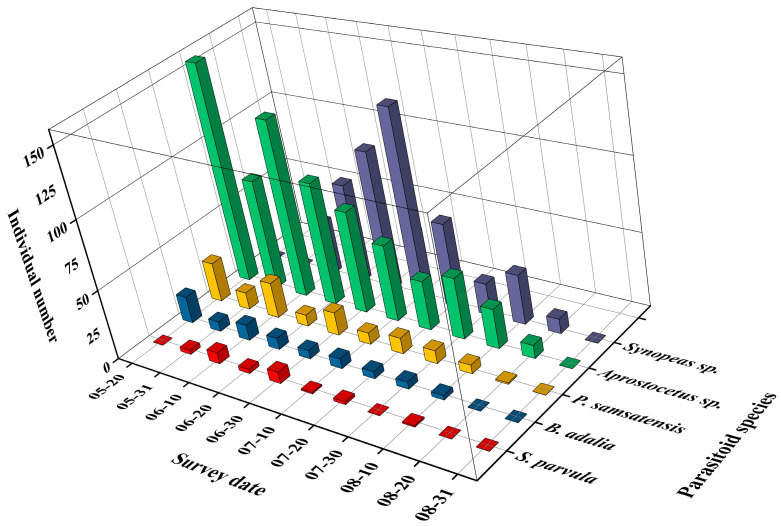
Dynamic analysis of parasitoid communities of *D*. *jujubifolia* in Hami, Xinjiang, China (2021).

The population dynamics of the parasitoids were closely aligned with the occurrence pattern of *D. jujubifolia*. Populations of *P. samsatensis*, *B. adalia*, and *Aprostocetus* sp. peaked simultaneously in mid-May. *S. parvula* was first detected in late May, peaked in late June, and disappeared by early August. *Synopeas* sp. first appeared in early June, with a peak in early July. The dominant parasitoid *P. samsatensis* exhibited four distinct population peaks during the study period—in mid-May, early June, late June, and mid-July. These peaks closely parallel host population dynamics, indicating high parasitism efficiency and close synchrony with host availability (Figure 6).

### 3.6. Emergence Patterns of D. jujubifolia Parasitoids Under Laboratory Conditions

**Figure 7:**
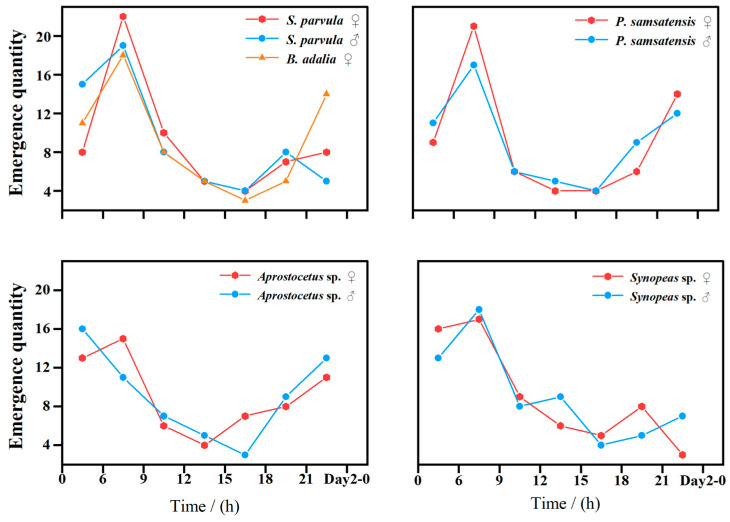
Daily emergence rhythms of parasitoids of *D*. *jujubifolia*.

Under laboratory conditions, parasitoids of *D. jujubifolia* emerged continuously across 24 h. Peak emergence occurred between 06:00–09:00 and 21:00–00:00. For *P. samsatensis*, 34% of individuals emerged between 06:00 and 09:00 and 22% between 21:00 and 00:00, with 16% overall emerging at night. *S. parvula* showed the highest emergence activity between 06:00 and 09:00, accounting for 37% of total emergence (20% females and 17% males), with 13% of males emerging at night. Female *B. adalia* also exhibited bimodal peaks at 06:00–09:00 and 21:00–00:00. In *Aprostocetus* sp., females predominantly emerged in the early morning, but a considerable proportion also emerged at night, whereas males peaked at 21:00–00:00. *Synopeas* sp. showed a single daytime peak at 06:00–09:00, accounting for 31% of total emergence, with 25% occurring at night. Overall, endoparasitoids exhibited higher nocturnal emergence than ectoparasitoids. These results improve our understanding of parasitoid emergence rhythms and provide practical guidance for optimizing field release timing to enhance the efficiency of biological control (Figure 7).

### 3.7. Adult Longevity and Sex Ratios of Parasitoids

Newly emerged adults were reared individually in glass tubes, with 30 females and 30 males tested per species under each feeding condition. Two-way ANOVA showed that adult longevity was significantly affected by species and feeding conditions, with significant species × feeding interactions in both sexes (Table 1). For females, effects were significant for species (F_4_, 290 = 32.29, *p* = 2.90 × 10^−22^), feeding conditions (F_1_, 290 = 303.52, *p* = 5.18 × 10^−47^), and their interaction (F_4_, 290 = 19.29, *p* = 4.39 × 10^−14^). For males (four species), species (F_3_, 232 = 33.91, *p* = 3.27 × 10^−18^), feeding conditions (F_1_, 232 = 209.55, *p* = 2.87 × 10^−34^), and their interaction (F_3_, 232 = 15.81, *p* = 2.18 × 10^−9^) were also significant. These results indicate species-specific responses to feeding conditions. *P*. *samsatensis* had the greatest longevity in both sexes, whereas *Synopeas* sp. had the shortest, with significant differences compared to the other species. Except for *B*. *adalia*, which reproduces parthenogenetically, all parasitoids exhibited female-biased sex ratios, with *Aprostocetus* sp. showing the highest female-to-male ratio (2.22).

## 4. Discussion

This study synthesizes the life history, developmental traits, damage symptoms, and parasitoid community of *D*. *jujubifolia* in Xinjiang, providing a solid foundation for gall-midge ecology and biological control [34,35]. In Hami, the species has 4–5 generations annually, consistent with northern China and Korea but differing from southern China (see Table S2 for climatic context) [10,36,37,38], indicating marked plasticity in niche use and life-history traits [39,40,41]. Temperature and humidity chiefly determine voltinism; in arid regions, water stress combined with the physiological status of host leaves further tightens the coupling between population dynamics and environmental constraints [42,43]. Difficulty completing the full life cycle under laboratory conditions underscores strong dependence on the gall microenvironment for moisture, nutrition, and enemy avoidance [44,45,46]. Future work should resolve gall chemistry and its roles in larval development and parasitoid host location, and identify volatile blends that reliably attract dominant parasitoids. In the field, pairing native flower/nectar strips with water-saving irrigation can extend parasitoid residency and sustain control. Avoid broad-spectrum insecticides during parasitoid activity peaks, and integrate selective insecticides with monitoring–early-warning systems to build a region-scale IPM framework centred on conservation biological control [47,48,49].

Comprehensive field surveys recorded five parasitoid species associated with *D*. *jujubifolia*, spanning the two principal functional groups—ectoparasitoids and endoparasitoids. Detailed morphological analyses confirmed the identities of *Systasis* sp. and *Pseudotorymus* sp., previously reported in Hebei Province, and provided updated descriptions with high-resolution images to support reliable identification [29]. Notably, *P*. *samsatensis* was the dominant species, showing strong synchrony with host generations and serving as the key regulatory factor in the field. From the perspective of host specificity, *P*. *samsatensis* and *B*. *adalia* are here reported for the first time as parasitoids of *D*. *jujubifolia*, with no records of other host associations to date, suggesting that both species may exhibit a high degree of host specialization. In contrast, *S*. *parvula* has been reported to parasitize several other cecidomyiid hosts, including *Bremiola caraganae* (Fedotova) (Diptera: Cecidomyiidae), *Dasineura heteroae* (Stelter) (Diptera: Cecidomyiidae), and *Schizomyia galiorum* Kieffer (Diptera: Cecidomyiidae), indicating a relatively broader host range [32]. Moreover, the genera to which these five parasitoid species belong are all known to include members associated with gall midges (Cecidomyiidae), implying an evolutionary tendency toward specialization on this host group [50,51,52,53]. Comparable patterns in other systems—e.g., *Systasis aceri* (Hymenoptera: Pteromalidae) on *Obolodiplosis robiniae* (Diptera: Cecidomyiidae) and *Platygaster* spp. on the European apple leaf gall midge—underscore the role of parasitoid diversity in natural regulation [54,55,56]. Functionally, the propensity to oviposit within enclosed gall structures likely enhances offspring survival under predator pressure. From an applied standpoint, we therefore prioritize *P. samsatensis* for augmentative use and targeted conservation; practical next steps include establishing stable colony-rearing protocols, quantifying female-biased release ratios, and using sentinel galls or emergence traps to calibrate phenology-based release windows and set decision thresholds. This study presents the first comprehensive characterization of the *D. jujubifolia* parasitoid community in Xinjiang, with two species newly recorded for China, providing taxonomic and biogeographic insights and laying a foundation for regionally adapted biological control strategies.

Extensive field investigations across more than 70 jujube orchards in Hami and surrounding areas confirmed that *D. jujubifolia* occurs ubiquitously in the region, with almost all trees showing gall formation on young leaves. Such high infestation levels clearly demonstrate that this species is a dominant pest in local jujube ecosystems. Although direct yield measurements were not conducted, interviews with orchard managers and local agricultural committees, together with previously published reports, indicate that heavy galling is associated with an estimated 20–30% reduction in fruit set in severely affected orchards [9]. These findings are consistent with previous studies and highlight the economic impact of this pest on jujube cultivation. Initially reported in China, *D. jujubifolia* has since spread to Korea, where it is recognized as an invasive species threatening local jujube production [10]. This cross-border spread underscores its potential as an emerging invasive pest of global concern. Given that jujube cultivation is expanding across multiple regions worldwide, particularly in arid and semi-arid zones, proactive quarantine and phytosanitary measures will be essential to prevent further spread [57,58]. In this context, the detailed morphological descriptions and high-resolution images of all developmental stages provided in this study are of practical value for plant protection and quarantine agencies. These diagnostic resources will facilitate accurate and rapid identification, strengthen early detection, and support exclusion efforts in countries where *D. jujubifolia* has not yet been recorded. Collectively, these findings underscore the urgent need for coordinated regional and international strategies integrating monitoring, quarantine, and ecological management to mitigate the risks associated with this pest’s invasion and establishment.

Compared with the jujube gall midge *D*. *jujubifolia* in Hami, Xinjiang (one generation ≈ 19–24 days), the blueberry gall midge *Dasineura oxycoccana* (Diptera: Cecidomyiidae) shows the most similar overall development time, requiring approximately 2–3 weeks from first-instar larva to adult at room temperature (complete generation ≈ 18–25 days). By contrast, *Dasineura pyri* (Diptera: Cecidomyiidae) completes a generation in ~25–30 days and *Dasineura mali* (Diptera: Cecidomyiidae) in ~35–40 days; larval feeding lasts ~2 weeks in *D. pyri* and ~2–4 weeks in *D. mali*, and both typically exhibit 3–4 overlapping generations per growing season. Despite these differences, overwintering is broadly similar: after the final generation leaves the foliage, individuals overwinter predominantly as larvae/pre-pupae within the upper 2–5 cm of soil [59,60,61,62]. At the applied level, based on the damage patterns and life history of *D*. *jujubifolia*, we propose three environmentally sustainable management strategies that are both feasible and broadly applicable, as follows: (1) systematically prune and destroy infested shoots before larvae enter the soil; (2) apply plastic mulch beneath the canopy before final-instar larvae move into the soil for overwintering, thus preventing entry into subterranean diapause; and (3) perform early spring tillage to expose pupae to surface conditions or bury them deeper, thereby enhancing natural mortality. These approaches are also applicable to the green management of other *Dasineura* pests, such as the apple leaf gall midge (*D. mali*), the pear leaf rolling midge (*D. pyri*), and the blueberry gall midge (*D. oxycoccana*), thereby contributing to the establishment of an integrated, systematic control framework for gall midges worldwide.

Although this study represents the first systematic characterization of *D*. *jujubifolia* and its parasitoid community in Xinjiang, several limitations remain. First, the survey was limited in temporal and spatial scope, restricting the comprehensive understanding of population dynamics across climatic regimes and broader geographic scales. Second, parasitoid community structure is likely shaped by cultivation practices, pesticide use, and vegetation context, highlighting the need for comparative analyses across larger spatial scales and more diverse ecosystems. Third, the ecological regulation measures proposed here, while of potential practical value, require long-term validation under field conditions to evaluate their effectiveness and economic feasibility. As concrete next steps, we recommend molecular tools to strengthen parasitoid identification and community profiling, including COI DNA barcoding, complementary nuclear markers (e.g., 28S rDNA, ITS2), and high-throughput metabarcoding of bulk gall/larval samples. Implementing these within an integrative taxonomy framework that links vouchers, morphology, and sequences—together with building a curated regional reference library—will reduce identification uncertainty and reveal cryptic diversity and succession. In parallel, chemical-ecology assays should test how parasitoids exploit plant volatiles and host cues for host location. In addition, developing pest–natural enemy dynamic models will allow the prediction of population trends under varying climate scenarios, thereby providing a stronger theoretical basis for designing regionally tailored, conservation-oriented management strategies.

## 5. Conclusions

This study identified five parasitoid species associated with *D. jujubifolia*, including two newly recorded in China: *P. samsatensis* and *B. adalia*. The dominant species, *P. samsatensis*, exhibited the highest parasitism rate and close synchrony with host generations, highlighting its key role in natural regulation. Integrating pest life history with parasitoid phenology, we propose environmentally sustainable management practices—including pruning, mulching, and spring tillage—that demonstrate strong feasibility and broad applicability. Overall, this study provides a scientific foundation for the conservation and utilization of parasitoids and offers new strategies and practical guidance for integrated pest management.

## Figures and Tables

**Table 1:** Adult longevity of parasitoid wasps under different nutritional conditions.

	Female (d)		Male (d)		Female–Male
	15% Honey	Water	15% Honey	Water	
*P. samsatensis*	10.80 ± 0.78 Aa	3.07 ± 0.21 Ba	7.07 ± 0.46 Ca	2.73 ± 0.17 Ba	1.38
*S. parvula*	6.53 ± 0.37 Ab	2.70 ± 0.20 Ba	5.63 ± 0.28 Ab	2.40 ± 0.17 Ba	1.56
*B. adalia*	9.73 ± 0.78 Aa	3.03 ± 0.19 Ba	/	/	/
*Aprostocetus* sp.	7.17 ± 0.49 Ab	2.90 ± 0.19 Ba	4.43 ± 0.25 Cc	2.43 ± 0.17 Ba	2.22
*Synopeas* sp.	3.03 ± 0.20 Ac	2.07 ± 0.14 Bb	2.87 ± 0.26 Ad	1.87 ± 0.13 Bb	1.63

Note: Values are means ± SE (*n* = 30 per sex per species). Different lowercase letters within the same feeding condition indicate significant differences among species, and different uppercase letters within the same species indicate significant differences between feeding conditions (Tukey’s HSD, *p* < 0.05). Two-way ANOVA statistics (F, df, *p*) are provided in the text.

## Data Availability

The original contributions presented in this study are included in the article/Supplementary Materials. Further inquiries can be directed to the corresponding authors.

## Supplementary Materials

The following supporting information can be downloaded at https://www.mdpi.com/article/10.3390/insects16111118/s1, Table S1. Developmental dynamics of *Dasineura jujubifolia* and parasitism rate, longevity, and occurrence dynamics of its parasitoids in Hami, Xinjiang, China (2021). Table S2. Monthly mean meteorological parameters in Hami, Xinjiang, China (April–September 2021).
